# Serum galectin‐3 levels and delirium among postpartum intensive care unit women

**DOI:** 10.1002/brb3.773

**Published:** 2017-07-21

**Authors:** Ying Zhu, Wei Hu, Ming‐Li Zhu, Ting Yin, Jun Su, Jian‐Rong Wang

**Affiliations:** ^1^ Department of Intensive Care Unit The Hangzhou First People's Hospital Nanjing Medical University Hangzhou China

**Keywords:** delirium, galectin‐3, intensive care unit, postpartum, women

## Abstract

**Objective:**

Inflammation correlates with delirium. Galectin‐3 is a proinflammatory protein. This study aimed to determine relation of serum galectin‐3 levels to delirium of postpartum intensive care unit (ICU) women.

**Materials and Methods:**

In this prospective observational study, serum galectin‐3, S100B, and C‐reactive protein levels of 412 postpartum ICU women and 412 healthy women were measured. Delirium and Acute Physiology and Chronic Health Care Evaluation II (APCHCE II) scores were recorded.

**Results:**

Serum levels of galectin‐3, S100B, and C‐reactive protein were significantly elevated in the postpartum women than in the healthy women. Galectin‐3 levels were highly associated with APCHCE II scores and S100B and C‐reactive protein levels. Galectin‐3, C‐reactive protein, and S100B levels and APCHCE II scores were identified as independent predictors for delirium. Area under the curve (AUC) of serum galectin‐3 levels was similar to that of S100B levels, and significantly exceeded those of C‐reactive protein levels and APCHCE II scores. Moreover, galectin‐3 significantly improved the AUCs of APCHCE II scores, S100B levels, and C‐reactive protein levels.

**Conclusions:**

Galectin‐3, involved in inflammatory process underlying delirium‐related brain injury, might be a potential biomarker to predict delirium of postpartum ICU women.

## 1 INTRODUCTION

Delirium is a common disorder in intensive care unit (ICU) patients, which is characterized by fluctuating changes in cognition, consciousness, and attention, prolongs ICU stay, increases mortality, and delays functional recovery markedly (Barbateskovic et al., 2016; Bhattacharya et al., 2017; Kratz, Heinrich, Schlauß, & Diefenbacher, 2015; Kumar, Jayant, Arya, Magoon, & Sharma, 2017; Lipowski, 1989; Sanguineti, Wild, & Fain, 2014). So far, actual pathophysiological mechanisms of delirium remain unclear, but a growing body of evidence indicates inflammation might play an important role (Cerejeira, Firmino, Vaz‐Serra, & Mukaetova‐Ladinska, 2010; Cerejeira, Nogueira, Luís, Vaz‐Serra, & Mukaetova‐Ladinska, 2012; Dillon et al., 2017). Galectin‐3, a member of the β‐galactoside‐binding galectin family, is uniquely chimeric (Ahmed & AlSadek, 2015; de Oliveira et al., 2015). Originally, galectin is found to be involved in multiple aspects, including cell adhesion, proliferation, clearance, apoptosis, cell activation, cell migration, and phagocytosis (Li, Li, & Gao, 2014; Peacock & DiSomma, 2014). Recently, it is identified as a proinflammatory protein and modulates immune responses either as damage‐associated molecular patterns or as pattern recognition receptors (Arad et al., 2015; Li et al., 2008; Zhang et al., 2015). Increased circulating levels of galectin‐3 are associated with inflammatory severity in some inflammatory diseases, for example, systemic lupus erythematosus, rheumatoid arthritis, heart failure, and systemic sclerosis (Koca et al., 2014; Nielsen et al., 2014; Ohshima et al., 2003; Piper, de Courcey, Sherwood, Amin‐Youssef, & McDonagh, 2016). Of note, activated glia can express galectin‐3 during neuroinflammation (Boza‐Serrano et al., 2014; Chen, Liao, Lin, & Liu, 2014; Sirko et al., 2015). Moreover, plasma galectin‐3 levels have close relation to inflammation, disease severity, and clinical outcome in traumatic brain injury, aneurysmal subarachnoid hemorrhage, and spontaneous intracerebral hemorrhage (Liu, Liu, Zhao, Liu, & He, 2016; Shen et al., 2016; Yan et al., 2016). Thus, galectin‐3 might reflect extent of brain injury and inflammatory status in some neurological diseases. Alternatively, postpartum ICU women are a group of critically ill patients. To date, galectin‐3 is not explored in postpartum women or patients with delirium. The present study aimed to investigate the ability of serum galectin‐3 levels to predict delirium in postpartum ICU women.

## 2 MATERIALS AND METHODS

### 2.1 Study population

This study included these women who were transferred to ICU within postpartum 24 hr and had ICU stays of more than 7 days from May 2014 to May 2016 in The Hangzhou First People's Hospital. The exclusion criteria included a documented history of dementia, delirium, or depressive illness, inability to speak or understand Chinese, any severe visual or auditory disorders, immunosuppressive disorders, neurological disorders, malignant disease, and chronic inflammatory disease. The control group consisted of healthy women who came to our hospital for healthy examination during the period of May 2015 to May 2016. We required that healthy women should be aged from 20 to 50 years old. The exclusion criteria were similar for the controls and the postpartum ICU women. This study was approved by the responsible committee on human experimentation in The Hangzhou First People's Hospital. Written informed consent was obtained from someone responsible for them.

### 2.2 Clinical assessment

Recorded information included age, pre‐pregnancy body mass index, medical comorbidity (i.e., congenital heart disease, severe acute pancreatitis, acute heart failure, and urolithiasis complicated with infection), obstetrical complications (i.e., obstetric hemorrhage, preeclampsia, gestational diabetes, and intrauterine infection complicated with sepsis), vasoactive agent use, mechanical ventilation, and blood purification. Disease severity was assessed using Acute Physiology and Chronic Health Care Evaluation II (APCHCE II) scores. Confusion Assessment Measurement for the ICU (CAM‐ICU) is the most widely used validated instrument to detect delirium in critical ill patients, both mechanically and not mechanically ventilated patients (Ely, Inouye, et al., 2001; Ely, Margolin, et al., 2001). The patients were assessed using CAM‐ICU every day until day 7 after ICU entry.

### 2.3 Immunoassay methods

Venous blood of patients and controls was drawn at entry into ICU and study, respectively. The blood samples were immediately placed into sterile test tubes and centrifuged at 3,000*g* for 30 min to collect serum, which was stored at −70°C until assayed. Serum galectin‐3, S100B, and C‐reactive protein levels were determined by enzyme‐linked immunosorbent assay using commercial kits (galectin‐3: BG Medicine, Inc., Waltham, MA, USA [catalog number: 12836]; S100B: R&D Systems, Minneapolis, MN, USA [catalog number: DY1820‐05]; C‐reactive protein: Cusabio Biotech, Wuhan, China [catalog number: CSB‐E08617h]) in accordance with the manufacturer's instructions. All samples were assayed in duplicate by the same laboratory technician having no access to all clinical data using the same equipment. Any samples, which had hemolysis, were excluded.

### 2.4 Statistical analysis

Statistical analysis was performed with SPSS 19.0 (SPSS Inc., Chicago, IL, USA) (RRID: SCR_002865) and MedCalc 9.6.4.0. (MedCalc Software, Mariakerke, Belgium) (RRID:SCR_015044). The categorical variables were presented as counts (percentage). Kolmogorov–Smirnov test or Shapiro–Wilk test was used to test the normality of data distribution for all continuous variables. In this study, all continuous data were non‐normally distributed and, subsequently, were reported as median (interquartile range). Comparisons were made using (1) chi‐square test or Fisher's exact test for categorical data, and (2) Mann–Whitney *U* test for continuous variables as appropriate. Bivariate correlation analyses between gelatin‐3 levels and other variables (i.e., S100B levels, C‐reactive protein levels, and APCHCE II scores) were conducted using Spearman's correlation coefficient. The independent predictors of delirium were investigated in a logistic regression model with estimate odds ratio (OR) and 95% confidence interval (CI). The receiver operating characteristic (ROC) curves were configured to determine the best threshold of the related predictors to identify the patients at risk of delirium. Subsequently, area under the curve (AUC) and the corresponding 95% CI were calculated. A combined logistic regression model was configured to estimate the additive benefit. A two‐tailed probability value of <.05 was considered as statistically significant.

This study contained comparison of two means and comparison of two ROC curves for serum galectin‐3 levels. We used sampling test in MedCalc software to calculate minimal required sample size. In terms of serum galectin‐3 levels, we selected 0.01 for both alpha and beta levels. For comparison of two means of serum galectin‐3 levels divided by delirium, minimal required sample size was 23. For comparison of two ROC curves based on serum galectin‐3 levels, minimal required sample size was 313. For comparison of two means of serum galectin‐3 levels between postpartum women and the controls, minimal required sample size was 10. Thus, in this study, minimal required sample size should be 313.

## 3 RESULTS

### 3.1 Study population characteristics

Initially, a total of 487 postpartum ICU women were assessed. According to the exclusion criteria, we excluded 5 women with a documented history of dementia, delirium, or depressive illness, 10 women who were unable to speak or understand Chinese, 2 women with a severe visual or auditory disorder, 15 women with an immunosuppressive disorder, 8 women with a neurological disorder, 4 women with malignant disease, 21 women with chronic inflammatory disease, 5 women whose samples had hemolysis, 3 women with incomplete information, and 2 women with unavailable samples. Finally, this study included a total of 412 postpartum ICU women. This group of postpartum ICU women had a mean age of 30.2 ± 6.4 years (range = 20–43 years). In addition, 412 healthy women were chosen as controls that had a mean age of 29.7 ± 6.7 years (range = 20–49 years). Thus, there was no statistically significant difference in age between controls and postpartum ICU women (*p *=* *.409).

Among postpartum women, the mean pre‐pregnancy body mass index was 24.2 ± 3.1 kg/m^2^ (range = 19.2–35.4 kg/m^2^). Medical comorbidities included congenital heart disease (40 patients, 9.7%), severe acute pancreatitis (21 patients, 5.1%), acute heart failure (20 patients, 4.9%), and urolithiasis complicated with infection (24 patients, 5.8%). With regard to obstetric complications, there were obstetric hemorrhage in 96 (23.3%) patients, preeclampsia in 70 (17.0%) patients, gestational diabetes in 45 (10.9%) patients, and intrauterine infection complicated with sepsis in 26 (6.3%) patients. After entry into ICU, vasoactive agent was used in 158 (38.4%) patients, 162 (39.3%) patients underwent mechanical ventilation, and blood purification was performed for 18 (4.4%) patients. Upon entry into ICU, there were a median APCHCE II score of 23 (9) (range = 4–39). Delirium was found in 85 (20.6%) patients.

### 3.2 Galectin levels and other variables

**Figure 1:**
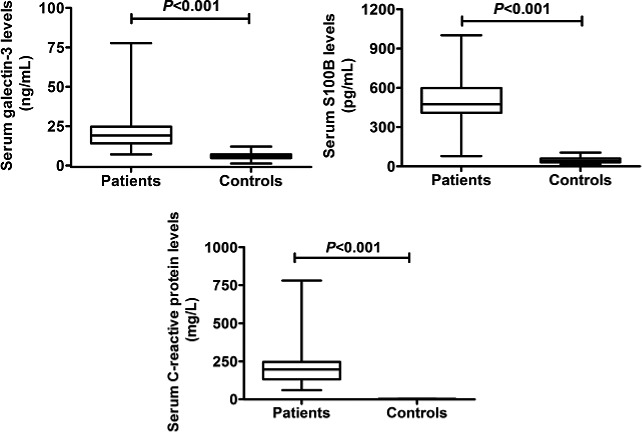
Comparisons of serum galectin‐3, S100B, and C‐reactive protein levels between the patients and controls. Data were reported as median (interquartile range) with maxima and minima

**Figure 2:**
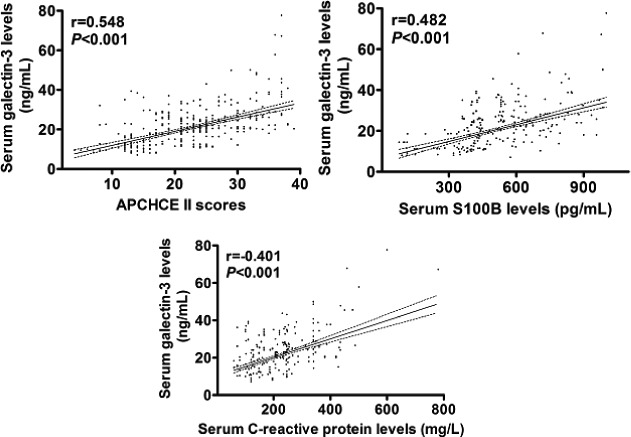
Correlative analyses of serum galectin‐3 levels with serum S100B and C‐reactive protein levels in addition to Acute Physiology and Chronic Health Care Evaluation II (APCHCE II) scores among postpartum intensive care unit women

In Figure 1, there were significant differences between the patients and controls in terms of serum galectin‐3, S100B, and C‐reactive protein levels. Also, in Figure 2, among the patients, serum galectin‐3 levels significantly correlated with serum S100B and C‐reactive protein levels in addition to APCHCE II scores.

### 3.3 Relation of galectin‐3 levels to delirium

Table 1 showed that the delirium patients had higher serum galectin‐3, S100B, and C‐reactive protein levels, higher APCHCE II score, as well as a higher percentage of congenital heart disease, severe acute pancreatitis, acute heart failure, urolithiasis complicated with infection, obstetric hemorrhage, preeclampsia, gestational diabetes, intrauterine infection complicated with sepsis, vasoactive agent use, mechanical ventilation, and requirement for blood purification. Furthermore, when the above variables, found to be significant in the univariate analysis, were introduced into the logistic model, APCHCE II score (OR, 1.171; 95% CI, 1.110–1.235; *p *=* *.002), S100B level (OR, 1.009; 95% CI, 1.007–1.012; *p *=* *.004), C‐reactive protein level (OR, 1.007; 95% CI, 1.004–1.011; *p *=* *.012), and galectin‐3 level (OR, 1.170; 95% CI, 1.116–1.226; *p *=* *.001) were identified as the independent predictors for delirium of postpartum ICU women.

**Table 1:** The factors related to delirium of postpartum women in intensive care unit

Parameter	Delirium	Nondelirium	*p* Value	OR (95% CI)	*p* Value
Number	85 (20.6%)	327 (79.4%)			
Age (years)	31.2 ± 5.8	30.0 ± 6.5	.097	1.030 (0.992–1.070)	.121
Body mass index (kg/m^2^)	24.8 ± 2.2	24.1 ± 3.3	.062	1.073 (0.996–1.156)	.064
Medical comorbidity					
Congenital heart disease	15 (17.7%)	25 (7.7%)	.006	2.589 (1.297–5.166)	.007
Severe acute pancreatitis	9 (10.6%)	12 (3.7%)	.022	3.109 (1.264–7.644)	.013
Acute heart failure	8 (9.4%)	11 (3.4%)	.036	2.985 (1.161–7.673)	.023
Urolithiasis complicated with infection	10 (11.8%)	14 (4.3%)	.016	2.981 (1.274–6.972)	.012
Obstetric complication					
Obstetric hemorrhage	30 (35.3%)	66 (20.2%)	.003	2.157 (1.282–3.630)	.004
Preeclampsia	25 (35.3%)	45 (13.8%)	.001	2.611 (1.487–4.584)	.001
Gestational diabetes	15 (17.7%)	30 (9.2%)	.026	2.121 (1.083–4.155)	.028
Intrauterine infection complicated with sepsis	11 (12.9%)	15 (4.6%)	.005	3.092 (1.364–7.008)	.007
Vasoactive agent use	43 (50.6%)	115 (35.2%)	.009	1.887 (1.166–3.056)	.010
Mechanical ventilation	44 (51.8%)	118 (36.1%)	.008	1.901 (1.174–3.077)	.009
Blood purification	7 (8.2%)	11 (3.4%)	.069	2.578 (0.968–6.866)	.058
APCHCE II score	30 (12)	21 (10)	<.001	1.220 (1.164–1.279)	<.001
Serum C‐reactive protein level (mg/L)	254 (113)	166 (114)	<.001	1.012 (1.009–1.015)	<.001
Serum S100B level (pg/ml)	688 (226)	437 (146)	<.001	1.010 (1.008–1.013)	<.001
Serum galectin‐3 level (ng/ml)	27 (12)	18 (9)	<.001	1.202 (1.152–1.254)	<.001

The categorical variables were expressed as counts (percentages). The continuous variables were reported as median (interquartile range). Proportions were compared using the chi‐square test or Fisher's exact test. The Mann–Whitney *U* test was used to compare the continuous variables between groups. The predictors of delirium were assessed using a univariate logistic regression analysis. The odds ratio (OR) values and 95% confidence intervals (CIs) were reported. APCHCE II indicates Acute Physiology and Chronic Health Care Evaluation II.

### 3.4 Discriminatory effect analysis for delirium

**Figure 3:**
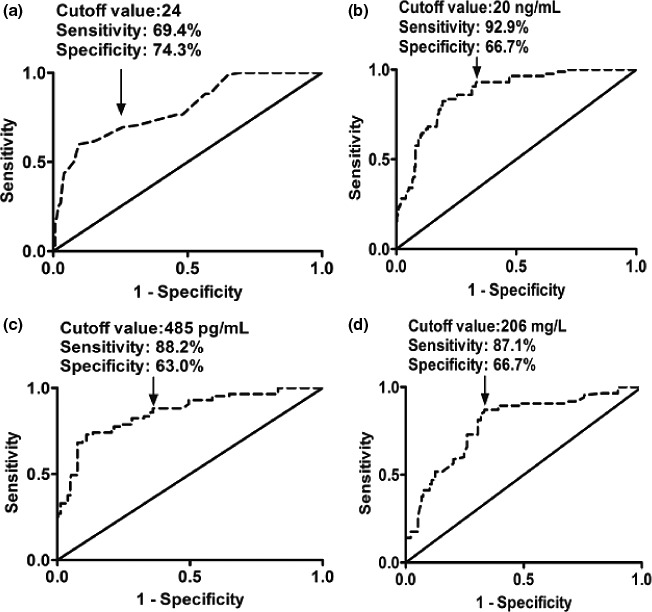
Receiver operating characteristic curve analyses of Acute Physiology and Chronic Health Care Evaluation II scores (a) as well as serum galectin‐3 (b), S100B (c), and C‐reactive protein levels (d) for delirium of postpartum intensive care unit women

In Figure 3, ROC curves demonstrated that serum S100B, C‐reactive protein, and galectin‐3 levels and APCHCE II scores predicted delirium with high AUCs. In addition, the predictive value of galectin‐3 levels was similar to that of S100B levels, and significantly exceeded those of C‐reactive protein levels and APCHCE II scores (Table 2). Moreover, just as shown in Table 2, the combined logistic regression model confirmed that galectin‐3 significantly improved the AUCs of APCHCE II scores, S100B levels, and C‐reactive protein levels.

**Table 2:** Receiver operating characteristic curve analyses for delirium among postpartum women in intensive care unit

	AUC (95% CI)	*p* Value	*p* Value	*p* Value
APCHCE II scores	0.800 (0.759–0.838)	Ref.		
S100B levels	0.856 (0.819–0.889)		Ref.	
C‐reactive protein levels	0.790 (0.747–0.828)			Ref.
Galectin‐3 levels	0.870 (0.834–0.901)	.023	.665	.021
APCHCE II scores combined with galectin‐3 levels	0.880 (0.844–0.910)	<.001		
S100B levels combined with galectin‐3 levels	0.921 (0.890–0.945)		.001	
C‐reactive protein levels combined with galectin‐3 levels	0.886 (0.851–0.915)			<.001

Under receiver operating characteristic curves, intergroup comparisons were done using *Z* test. The area under the curve (AUC) values and 95% confidence intervals (CIs) were reported. APCHCE II indicates Acute Physiology and Chronic Health Care Evaluation II; Ref., reference.

## 4 DISCUSSION

To the best of our knowledge, the current study, for the first time, investigated serum galectin‐3 levels in delirium patients. We enrolled a total of 412 postpartum ICU women, whose sample size was larger than the preceding calculated 313, and therefore, this study had enough statistical power to demonstrate that (1) serum galectin‐3 levels were significantly elevated in the postpartum ICU women than in the healthy women and in the delirium postpartum ICU women than in the nondelirium ones; (2) galectin‐3 in serum emerged as an independent predictor for delirium among postpartum ICU women; and (3) serum galectin‐3 levels showed high predictive value based on ROC curve. These results indicate that galectin‐3 might be a good predictor to identify the postpartum ICU women at risk of delirium.

Delirium can be caused by various causes, including sepsis, trauma, surgery, stress, and shock (Afonso et al., 2010; Bruce, Ritchie, Blizard, Lai, & Raven, 2007; de Jonghe et al., 2007; Koster, Oosterveld, Hensens, Wijma, & van der Palen, 2008; Tan et al., 2008). Postpartum ICU women are often complicated with multiple illnesses, for example, acute pancreatitis, acute heart failure, urolithiasis, infection, hemorrhage, hypertension, and mellitus diabetes (Aarvold et al., 2017; Igbaruma et al., 2016; Jonard, Ducloy‐Bouthors, & Fourrier, 2016), and therefore such a group of patient is susceptible to delirium. In this study, incidence of delirium within 7 days after ICU entry was 20.6% among postpartum women. Although the mechanisms underlying delirium were unknown, the accumulating evidence indicates that brain injury (Hall et al., 2013; van Munster, Bisschop, et al., 2010; van Munster, Korevaar, et al., 2010; van Munster et al., 2009) and inflammation (Cerejeira et al., 2010, 2012; Dillon et al., 2017) might be involved in this process. Consistent with this statement, we also found that serum S100B and C‐reactive protein levels were obviously elevated in postpartum ICU women, especially in delirium women. Thus, inflammation and secondary brain injury actually play a crucial role in the pathogenesis of delirium.

Delirium is associated with increased mortality and poor functional recovery (Kratz et al., 2015; Lipowski, 1989; Sanguineti et al., 2014). Hence, early prognostication of delirium would be beneficial to optimize treatment. S100B reflects extent of brain injury, and C‐reactive protein is a marker of inflammation. A growing body of evidence has shown that S100B and C‐reactive protein levels in peripheral blood can identify patients at risk of delirium (Dillon et al., 2017; Hall et al., 2013; van Munster, Bisschop, et al., 2010; van Munster, Korevaar, et al., 2010; van Munster et al., 2009). The current study also demonstrated that serum S100B and C‐reactive protein levels were the independent predictors for delirium among postpartum women in ICU. Undoubtedly, APCHCE II score, a determinant of severity in critical illnesses, was identified as the predictor for delirium in such a group of postpartum women. Intriguingly, serum S100B and C‐reactive protein levels and APCHCE II score showed the high predictive value in accordance with ROC curve. Therefore, serum S100B and C‐reactive protein levels and APCHCE II scores have the potential to be the good predictors for delirium among postpartum ICU women.

Galectin‐3 is reported to be involved in inflammation (Koca et al., 2014; Nielsen et al., 2014; Ohshima et al., 2003; Piper et al., 2016). Recently, many reports have shown that serum galectin‐3 levels were highly associated with severity and clinical outcomes of some brain injury diseases, including traumatic brain injury, intracerebral hemorrhage, and spontaneous subarachnoid hemorrhage (Liu et al., 2016; Shen et al., 2016; Yan et al., 2016), indicating that galectin‐3 might be a good biomarker for neurological diseases. This study confirmed that serum galectin‐3 levels were significantly elevated in postpartum ICU women, especially in delirium women, and moreover, its serum levels were correlated with serum S100B and C‐reactive protein levels as well as APCHCE II scores. These data indicate that serum galectin‐3 levels might have close relation to inflammation and brain injury underlying delirium among postpartum ICU women.

In the current study, besides serum S100B and C‐reactive protein levels in addition to APCHCE II scores, galectin‐3 in serum was demonstrated to be an independent predictor for delirium of postpartum women in ICU. Interestingly, we found that, compared to serum S100B levels, serum galectin‐3 levels possessed the similar predictive ability for delirium based on AUC; notably, its predictive ability significantly exceeded those of serum C‐reactive protein levels and APCHCE II scores. Importantly, serum galectin‐3 levels statistically significantly improved AUCs of serum S100B and C‐reactive protein levels in addition to APCHCE II scores. Thus, serum galectin‐3 levels may help to discriminate ICU women at risk of postpartum delirium.

## 5 CONCLUSIONS

In this study, elevated galectin‐3 levels correlate with serum S100B and C‐reactive protein levels as well as APCHCE II scores. Galectin‐3 in serum is demonstrated to independently predict delirium among postpartum women in ICU and has high discriminatory ability for delirium based on ROC curve. Thus, galectin‐3 may be involved in inflammatory process underlying delirium‐related brain injury and have the potential to be a biomarker to predict delirium of postpartum ICU women.

## CONFLICTS OF INTEREST

The authors have no conflict of interest.
